# Progression in Time of Dentate Gyrus Granule Cell Layer Widening due to Excitotoxicity Occurs along In Vivo LTP Reinstatement and Contextual Fear Memory Recovery

**DOI:** 10.1155/2022/7432842

**Published:** 2022-09-27

**Authors:** Karina Hernández Mercado, Araceli Martínez Moreno, Luis Francisco Rodríguez Durán, Martha L. Escobar, Angélica Zepeda

**Affiliations:** ^1^Departamento de Medicina Genómica y Toxicológica Ambiental, Instituto de Investigaciones Biomédicas, Universidad Nacional Autónoma de México, Ciudad de México, Mexico; ^2^División de Investigación y Estudios de Posgrado, Facultad de Psicología, Universidad Nacional Autónoma de México, Ciudad de México, Mexico

## Abstract

The dentate gyrus (DG) is the gateway of sensory information arriving from the perforant pathway (PP) to the hippocampus. The adequate integration of incoming information into the DG is paramount in the execution of hippocampal-dependent cognitive functions. An abnormal DG granule cell layer (GCL) widening due to granule cell dispersion has been reported under hyperexcitation conditions in animal models as well as in patients with mesial temporal lobe epilepsy, but also in patients with no apparent relation to epilepsy. Strikingly, it is unclear whether the presence and severity of GCL widening along time affect synaptic processing arising from the PP and alter the performance in hippocampal-mediated behaviors. To evaluate the above, we injected excitotoxic kainic acid (KA) unilaterally into the DG of mice and analyzed the evolution of GCL widening at 10 and 30 days post injection (dpi), while analyzing if KA-induced GCL widening affected in vivo long-term potentiation (LTP) in the PP-DG pathway, as well as the performance in learning and memory through contextual fear conditioning. Our results show that at 10 dpi, when a subtle GCL widening was observed, LTP induction, as well as contextual fear memory, were impaired. However, at 30 dpi when a pronounced increase in GCL widening was found, LTP induction and contextual fear memory were already reestablished. These results highlight the plastic potential of the DG to recover some of its functions despite a major structural alteration such as abnormal GCL widening.

## 1. Introduction

The dentate gyrus (DG) is the hippocampal subfield through which sensory information coming from the entorhinal cortex (EC) enters the hippocampus [1]. Granule cells from the DG are activated by excitatory synaptic inputs from the entorhinal cortex via the perforant pathway (PP) [2, 3]. The adequate DG function is critical for information processing coming from the PP and for the execution of hippocampal-dependent cognitive behaviors [4]. Importantly, the DG is particularly susceptible to suffer structural alterations under pathological hyperexcitation conditions [5]. An abnormal granule cell layer (GCL) widening due to granule cell dispersion (GCD) has been reported to occur within the normal spectrum of anatomical variation [6] but also in patients with mesial temporal lobe epilepsy and in sudden unexplained deaths in infants [7, 8]. In this condition, granule cell bodies are abnormally separated and ectopically located within the molecular layer provoking GCL widening [7].

The intrahippocampal injection of kainic acid (KA), a glutamate agonist, reproduces most of the alterations in the DG observed under pathological hyperexcitation conditions [5]. It has been reported that CGL widening due to GCD gradually develops after seizure activity of long duration early after intrahippocampal KA injection in mice [9]. Some studies suggest that GCD is involved in seizure generation and cognitive alterations given that the ectopic location of granule cells may represent a substrate of recurrent excitation [10, 11]. However, findings from other studies show that granule cells surviving in zones of GCD downscale their intrinsic excitability, mainly due to an increase in K^+^ conductance [12, 13]. This intrinsic rescaling of granule cells has been suggested to represent an adaptive mechanism in GCD zones that likely allows counterbalancing hyperexcitability thus maintaining some DG functions under pathological conditions [14].

We have previously reported that a subepileptogenic injection of KA directly into the upper DG blade of adult rats leads to a focal lesion, which shrinks in time. The decrease in the volume of the focal lesion occurs along the recovery of DG behavioral and synaptic functions. However, in our rat model, GCL widening due to GCD was not a salient feature [15, 16]. Therefore, whether DG function and hippocampal-mediated cognition are affected by the presence and severity of GCL widening along time is yet to be clarified. In this study, we aimed at evaluating to what extent does the presence and severity of KA-induced GCL widening impact on DG information processing arising from the PP and on hippocampal-mediated cognitive behaviors. To this end, we infused KA unilaterally into the upper blade of the mouse DG, where GCL widening has been shown to be consistently induced [5], and we analyzed the time course of GCL widening development and its association with long-term potentiation induction and contextual fear memory performance. We hereby show that GCL widening increases in time while DG functions become reinstated. Our results highlight the plastic potential of the DG as some initially lost functions due to excitotoxicity are recovered despite structural alterations such as GCL widening.

## 2. Materials and Methods

### 2.1. Animals

Adult 8-week-old C57BL/6 male mice were used throughout the experiment. Mice were housed four to five per cage with free access to water and food and were kept in an inverted 12 h artificial light/dark cycle under standard conditions. All animal procedures were performed in agreement with local government rules (Official Mexican Standard NOM-062-ZOO-1999). Experimental protocols were approved by the animal care committee at the Instituto de Investigaciones Biomédicas-Universidad Nacional Autónoma de México (UNAM). Efforts were made to minimize animal suffering and to reduce the number of subjects used.

### 2.2. Experimental Groups

For surgery and further behavioral analysis, we used an original *n* = 51. For behavioral analysis, our final *n* = 43 as some animals did not meet the criteria for inclusion (see below). During experimentation, mice were randomly assigned to the following groups: intact (*n* = 8), 10 days post sham surgery (10 dsh; *n* = 9), 30 days post sham surgery (30 dsh; *n* = 8), 10 days post-KA injection surgery (10 dpi; *n* = 9), and 30 post-KA injection surgery (30 dpi; *n* = 9). For histological analysis, we performed the measurements as follows: intact (*n* = 3), 10 dsh (*n* = 5), 30 dsh (*n* = 5), 10 dpi (*n* = 6), and 30 dpi (*n* = 5); all subjects included in the histological evaluation underwent behavioral assessment. For in vivo electrophysiological recordings in the perforant pathway, independent mice (original *n* = 26; final *n* = 20) not exposed to behavioral testing were used and assigned during experimentation to groups: intact (*n* = 4), 10 dsh (*n* = 4), 30 dsh (*n* = 4), 10 dpi (*n* = 4), and 30 dpi (*n* = 4).

#### 2.2.1. Experimental Design

**Figure 1:**
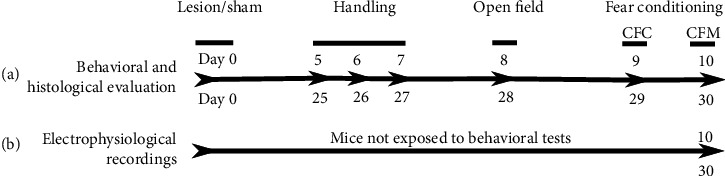
Experimental design. Kainic acid injections or sham surgeries were performed on day 0. Mice were handled and habituated to the experimenter for three consecutive days before the onset of behavioral procedures. On days 8 or 28 after surgery, mice performed the open field task. The next day (day 9 or 29), mice underwent contextual fear conditioning (CFC), and 24 h later, contextual fear memory (CFM) was evaluated. After memory retrieval, subjects were sacrificed and brains were extracted for histological analysis. A different group of animals, not exposed to behavioral evaluation, was used for in vivo LTP recordings.

Kainic acid injections or sham surgeries were performed on day 0. Mice were handled and habituated to the experimenter for three consecutive days before the onset of behavioral procedures. On days 8 or 28 after surgery, mice performed the open field task. The next day (day 9 or 29) mice underwent contextual fear conditioning (CFC), and 24 h later, contextual fear memory (CFM) was evaluated. Immediately after memory retrieval, subjects were sacrificed and brains were extracted for histological analysis. A different group of animals, not exposed to behavioral evaluation, was used for in vivo LTP recordings (Figure 1).

### 2.3. Stereotaxic Injection of KA and Induction of DG Excitotoxic Damage

We induced a focal excitotoxic lesion in the DG through the unilateral administration of a kainic acid (KA) solution. Each mouse was anaesthetized with 2–3% isoflurane in a mixture of 95% O_2_/5% CO_2_ and placed in a stereotaxic system (Stoelting, Wood Dale, IL). The scalp was disinfected, incised, and retracted to expose the skull. The right dorsal DG of the hippocampus was located relative to the bregma: anteroposterior (AP), −2 mm; mediolateral (ML), 1.3 mm; and dorsoventral (DV), –2 mm. A circular craniotomy was performed in the mentioned coordinates, and the dura was carefully sliced with the tip of a syringe needle to allow the cannula insertion. We injected 0.15 *μ*l of a 0.75 mM KA solution (see, kainic acid solution; Sigma–Aldrich, Chemie, St. Louis, MO) at a rate of 0.15 *μ*l/min through a 10 *μ*l Hamilton microsyringe mounted on a microinjection pump (Stoelting, Co., Wood Dale, IL, USA). For the sham group, the cannula was placed in the same coordinates under identical surgical conditions but no solution was delivered. In both cases, the cannula was left in the place for two min and was then slowly withdrawn; this prevented the reflux of the KA solution. The skin was sutured, and anesthesia was discontinued. Following recovery from anesthesia, mice were kept under observation for 3–4 hours. During this period, approximately, 70% of mice receiving KA injection experienced behavioral status epilepticus characterized by convulsive movements, rotations, and immobility as previously described [17] while all animals continued in the experimental protocol. No drugs were administrated after KA infusion to ameliorate behavioral status epilepticus, and no mortality was observed in the present study. The animals were left in their respective acrylic cages until behavioral testing or electrophysiological recordings were performed. Animals in which we could not distinguish the injection site and which did not develop GCD were left out of the analysis.

### 2.4. Kainic Acid Solution

Kainic acid (KA) (K0250; Sigma–Aldrich, Chemie, St. Louis, MO Sigma–Aldrich, MO) was dissolved in 1 N NaOH, and the solution was brought to the desired volume with 10 mM phosphate buffer (pH 7.2) for a final 0.75 mM solution. The pH was adjusted to 7.0 with 2 N HCl. A fluorescence tracer (red retrobeads, Lumafluor, Naples, FL) was added to the stock KA solution ((49 *μ*l of KA (0.75 mM) + 1 *μ*l of fluorescence tracer) for proper visualization of the injection site and KA spreading within the GCL.

### 2.5. Histological Procedures

Mice were deeply anesthetized with an overdose of sodium pentobarbital (210 mg/kg) and transcardially perfused with ice-cold 0.9% saline solution followed by ice-cold 4% paraformaldehyde in 0.1 M phosphate buffer (pH 7.4). Next, brains were removed, postfixed in paraformaldehyde for 24 h, and then successively transferred to 15% and 30% sucrose in 0.1 M phosphate buffer. We obtained 40 *μ*m thick coronal brain sections using a cryostat (Microm HM550, Thermo Fisher Scientific, Waltham, MA, USA). One in every three sections was mounted on gelatin-covered slides for Nissl staining.

### 2.6. Nissl Staining

Nissl staining was performed to analyze the gross morphology of the DG in all groups. The DG volume, as well as the volume of the focal lesion and the GCL width from the injected DG, were measured. Sections were mounted on gelatin-covered slides and rehydrated with distilled water. Then, sections were dehydrated in a series of EtOH solutions (70, 95, and 100%, 2 min in each) and immersed in xylol. Sections were then rehydrated, immersed in 0.1% cresyl violet (Sigma–Aldrich, St. Louis, MO, USA) and dehydrated before being covered with Permount (Fischer Scientific, NJ, USA) and a coverslip. Sections were captured with a 20x objective using a disk scanning unit microscope (Olympus BX51WI).

### 2.7. Measurement of the GCL Width

To evaluate the impact of the KA injection on GCL widening, the average width of the GCL was determined from Nissl-stained sections in all experimental groups following the procedures described in [18] using the Stereo Investigator software (MBF Bioscience Inc., Williston, VT).

Briefly, the brain section showing the maximal GCL widening from the dorsal DG ipsilateral to the injection was chosen as a start point for the measurements. We analyzed 4–5 consecutive sections from the “start section” (each section separated by 80 *μ*m from the next) and measured the GCL width (in the dorsoventral plane) at regular 100 *μ*m intervals in the mediolateral (ML) axis along the upper and lower blades from each section. In all cases, a total length of 520 *μ*m in the anteroposterior axis was analyzed. For calculating the dimension of the focal lesion zone produced in the upper blade, measurements were performed at shorter intervals of 50 *μ*m (in the mediolateral axis) due to the reduced blade area. For statistical analysis, the mean width from each DG-analyzed section and region (i.e., upper blade, lower blade, and lesion area) was calculated.

### 2.8. Volumetric Analysis of the DG and the Focal Lesion Zone

The volume of the dentate granule cell layer from Nissl-stained sections was calculated using the Cavalieri estimator stereological method (Stereo Investigator software; MBF Bioscience Inc., Williston, VT) as previously described [15]. Six sections per brain were used; each one was separated from the next by 80 *μ*m within AP −1.94 mm and −2.46 mm. The contour of the DG from all sections was delineated, the volume of the granule layer from each subject was automatically calculated, and the average per group was obtained and reported in mm^3^. The volume of the focal lesion zone produced in the upper blade was obtained using the same method by delineating the thinned area in five sections per brain.

### 2.9. In Vivo Electrophysiological Recordings

#### 2.9.1. Long-Term Potentiation (LTP) in the PP-DG

Mice were anesthetized with pentobarbital (50 mg/kg), and their temperature was maintained with gel thermal pads. Mice were fixed on a stereotaxic frame, and the skull was exposed. Unilateral responses were registered with a stainless-steel monopolar electrode located in the dentate gyrus (DG) in the coordinates: AP −2.0 from the bregma, ML −1.2 from the midline, and DV −1.8 from the dura [19, 20]. Responses were unilaterally evoked by a bipolar concentric stainless-steel stimulation electrode (100 *μ*m diameter) located in the perforant pathway (PP) at the following coordinates: AP −0.2 from Lambda, ML −2.8 from the midline, and DV −1.5 from the dura. Constant (400–800 *μ*A monophasic pulse, 0.25 ms) duration current stimulation was provided by a Grass S48 stimulator and delivered to the stimulating electrode through a Grass Stimulus Isolation Unit (PSUI6). Evoked responses were sent to an amplifier Grass P5. The electric signal was digitalized, stored, and analyzed using the software Datawave SciWorks (Broomfield, CO, USA). Baseline was established after 20 min of evoked responses. The applied stimulus intensity corresponded to 50% of the excitatory postsynaptic potential (EPSP) maximum amplitude. LTP was induced by delivering 5 high-frequency conditioning stimulus trains, each consisting of 10 bursts of 20 pulses each (400 Hz with 15 s of intertrain time [20].

### 2.10. Behavioral Testing

The open field task was conducted on days 8 or 28 post-KA injection or sham procedures to analyze anxiety and general motor performance. This test was followed by contextual fear conditioning (CFC) on days 9 or 29 and contextual fear memory (CFM) on days 10 or 30 after surgical procedures (see Figure 1). Mice were handled and habituated to the experimenter for three consecutive days before behavioral procedures were performed. Thirty minutes before behavioral evaluation, mice were habituated to the testing room.

### 2.11. Open Field Test

Mice were evaluated in the open field arena to analyze anxiety and general motor performance one day before CFC. The open field consisted of a white acrylic arena, 40 cm width × 40 cm length × 25 cm height. To facilitate the analysis of locomotion and the time spent at the center and periphery zones, the floor was divided into 16 drawn squares (10 cm × 10 cm each square) where 4 were central and 12 were peripheral. Each mouse was individually placed in a corner of the arena and was allowed to explore freely. Behavior was videotaped for 5 minutes. The total number of line crossings (from one square to another), the time spent at the center or periphery zones, and the displacement track from each mouse were manually assessed by the experimenter through repeated analysis of videos recorded during the test sessions. Caution was taken to counterbalance the introduction of the mice to each of the four corners of the arena. Before every trial, the arena was wiped with a cleaning solution consisting of 10% EtOH and 10% Extran (Merck, Darmstadt, Germany) diluted in distilled water.

### 2.12. Contextual Fear Conditioning and Memory

The task was performed in a mouse-conditioning chamber measuring 25 cm × 25 cm in length and 20 cm height (San Diego Instruments, San Diego, CA, USA). The chamber consisted of transparent acrylic walls and 23 stainless steel floor rods. The system was equipped with a matrix of 16 × 16 infrared beams at the floor level. Movements inside the chamber were registered by the interruption of a beam and were recorded with the aid of the freeze monitor software (Freeze Monitor, SD Instruments). Movements were also recorded by an observer and were compared to those recorded by the computer. The contextual fear conditioning (CFC) phase was performed on day 9 or 29 after KA injection or after sham surgery. Mice were placed individually in the conditioning chamber and were allowed to explore it for 2 min before the onset of the first foot shock. After this lapse, four foot shocks (2 s, 0.5 mA) were delivered at variable intervals ranging from 30 to 180 seconds. The preshock interval lasted 120 s, the postshock time interval from the first to the second shock was 150 s (first trial), from the second to the third was 90 s (second trial), from the third to the fourth was 120 s (third trial), and from the fourth to the end was 180 s (fourth trial). The percentage of freezing time was measured during the whole interval from one shock to the next. Only mice that responded to all foot shocks with jumps or vocalizations in all conditioning trials were included in the study. Twenty-four hours later, subjects were evaluated for contextual fear memory by placing each mouse in the conditioning chamber in the absence of foot shock and their freezing time was recorded for 5 min. Subjects were then returned to their home cages. Before each session, the chamber was wiped with a cleaning solution consisting of 10% EtOH and 10% Extran (Merck, Darmstadt, Germany) diluted in distilled water.

An experimenter, aware of the group conditions, calculated the percentage of freezing time reported by the freeze monitor software and quantified twice each parameter evaluated in the open field test while playing each video at a slow speed. However, as a measure of observational control, a different experimenter, blinded to the group conditions, randomly reevaluated the open field videos and calculated the percentage of freezing from the results generated by the freeze monitor software. Inter-rater correlation coefficient was 0.88.

### 2.13. Statistical Analysis

Data were analyzed using GraphPad Prism 7 software. One-way ANOVA followed by a Bonferroni's post hoc test was used to analyze data from the following: the Cavalieri estimation of the DG volume, the number of line crossings in the open field, and immobility time for the contextual fear memory task. An unpaired *t*-test with Welch's correction was used to analyze the Cavalieri estimation of the lesion volume in the DG upper blade and to analyze the obtained measurements of the DG width in the upper and lower blades. Two-way ANOVA followed by a Bonferroni's post hoc test was performed to analyze the time spent at the periphery and center zones in the open field and time of immobility during contextual fear conditioning. A repeated measures ANOVA followed by post hoc Fisher's test was used to analyze LTP results. Data evaluated through ANOVA passed the normality and equality variance tests. *p* < 0.05 was considered statistically significant in all cases.

## 3. Results

### 3.1. Morphological Changes over Time in the DG after a Focal Injection of KA

**Figure 2:**
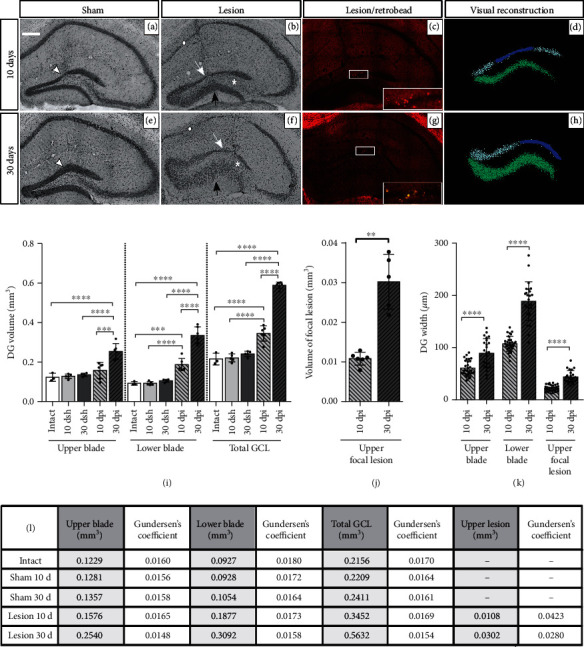
Morphological changes in the DG over time after KA injection. Left images show representative Nissl-stained coronal sections of the hippocampus from (a) 10 dsh, (b) 10 dpi, (e) 30 dsh, and (f) 30 dpi. (a) At 10 dsh, sections display a mechanical lesion in the upper blade of DG as result of the cannula insertion (head arrow); scale bars: 300 *μ*m. (e) At 30 dsh, the mechanical lesion is no longer evident (head arrow). (b) At 10 dpi, a focal lesion zone in the upper blade characterized by a thinning of the granule cell layer is observed (white arrow). Outside this lesion area, the upper blade shows GCL widening. (f) At 30 dpi, both DG blades display a more severe widening compared to 10 dpi. The black arrows indicate the prominent widening in the lower blade. The white circle and the asterisk symbols indicate cell loss in CA1 and in the hilus, respectively. (c–g) The site where KA spreads in the upper blade as revealed by the fluorescent tracer; insets show 40x magnifications. (d–h) A visual model of the DG based on Nissl-stained coronal sections: upper blade in gray, lesion site in blue, and lower blade in green. (i) Volume of the DG based on Cavalieri analysis (intact *n* = 3; 10 dsh *n* = 5; 30 dsh *n* = 5; 10 dpi *n* = 6; 30 dpi *n* = 5; one-way ANOVA followed by Bonferroni's post hoc test, *F*_(14, 60)_ = 123.7, *p* < 0.0001). (j) Volume from the lesion site in the upper blade from KA-injected mice (10 dpi *n* = 6; 30 dpi *n* = 5; unpaired *t*-test with Welch's correction, *F*_(4, 5)_ = 18.31, *p* < 0.0069). (k) GCL width from each DG section analyzed in KA-injected mice (10 dpi *n* = 29 sections from 6 mice; 30 dpi *n* = 24 sections from 5 mice; unpaired *t*-test with Welch's correction; upper blade, *F*_(23, 28)_ = 3.625, *p* = 0.0015; lower blade, *F*_(24, 28)_ = 6.493, *p* < 0.0001; upper blade focal lesion, *F*_(23, 28)_ = 3.962, *p* = 0.0007). (l) Average volume and Gundersen's coefficient after a stereological Cavalieri analysis from the same mice used in histograms (i, j); statistical significances are indicated in histograms (i, j). A Gundersen's coefficient less than 0.1 means statistically valid volume of data. The columns represent the mean SD from each group. Dots superimposed in the graph represent individual values. Asterisks indicate statistically significant differences (^∗^*p* < 0.05; ^∗∗^*p* < 0.01; ^∗∗∗^*p* < 0.001; ^∗∗∗∗^*p* < 0.0001).

We injected KA unilaterally in the upper blade of the dorsal DG and induced a focal excitotoxic lesion in the DG along with GCL widening. In order to analyze the evolution of the KA injection upon the DG, we evaluated the gross morphological changes of the DG blades as well as the evolution of the focal lesion at 10 and 30 days post-KA injection (dpi; Figure 2). The DG of sham mice at 10 days post sham surgery (10 dsh) showed mechanical damage provoked by the cannula insertion in the upper blade (Figure 2(a), arrowhead) which was not evident in the 30 dsh group (Figure 2(e), head arrow). None of the sham groups showed GCL widening (Figures 2(a) and 2(e)). However, a progressive increase in GCL widening was observed from 10 dpi to 30 dpi in the KA injection groups (Figures 2(b) and 2(f)). Figure 2(k) shows a wider GCL at 30 dpi than at 10 dpi in the upper (10 dpi: (mean ± SD) 60.64 *μ*m ± 14.63 < 30 dpi: 89.03 *μ*m ± 27.85) and lower blades (10 dpi: 107.3 *μ*m ± 14.58 < 30 dpi: 188.8 *μ*m ± 37.16), as well as in the upper focal lesion site (10 dpi: 22.37 *μ*m ± 5.96 < 30 dpi: 43.84 *μ*m ± 11.88). GCL widening consisted of the increase in the volume and width of both DG blades due to granule cell distant apposition (Figures 2(i) and 2(k), 10 dpi < 30 dpi, *p* < 0.0001). Still, in the lower blade, this effect was consistently stronger than that in the upper blade where the KA-induced focal lesion was located (Figure 2(i) volume, 30 dpi: upper (mean ± SD) 0.2536 mm^3^ ± 0.03984 vs lower 0.3352 mm^3^ ± 0.04337, *p* = 0004, and Figure 2(k), 10 dpi: upper 60.64 *μ*m ± 14.63 vs lower 107.3 *μ*m ± 14.58, 30 dpi: upper 80.03 *μ*m ± 27.85 vs lower 188.8 *μ*m ± 37.16, *p* < 0.0001). Along the septotemporal axis, we observed that in septal sections, GCD completely covered both DG blades (~AP: −2.06 to −2.54 mm from the bregma). However, in most temporal sections, GCD was visible only in the top part of the DG (~AP: −3.16 to −3.52 from the bregma; ~DV: −2.12 to −2.75 mm) and in the ipsi but not in the contralateral injected DG (Supplementary Figure 1, 2 and 3).

At 10 dpi, the KA-induced focal lesion was characterized by a thinning of the granule cell layer due to cell loss (decreased layer volume and width), but at 30 dpi, the lesion site had widened probably as a result of the dispersion of the surviving granule cells (Figures 2(b) and 2(f), white arrow; blue-colored zone in Figures 2(d) and 2(h); volume in Figure 2(j), 10 dpi: 0.01083 mm^3^ ± 0.00162 vs 30 dpi: 0.03024 mm^3^ ± 0.00695, *p* < 0.0027; width of focal lesion in Figure 2(k), 10 dpi: 22.37 *μ*m ± 5.97 vs 30 dpi: 43.84 *μ*m ± 11.88, *p* < 0.0001). This KA-induced focal lesion colocalized with the area where the KA spread, as revealed by the fluorescent tracer (Figures 2(c) and 2(g)). The volume of the DG blades was similar across time between sham and intact groups (Figure 2(i), total GCL in intact: 0.2156 mm^3^ ± 0.0295, 10 dsh: 0.2209 mm^3^ ± 0.0199, 30 dsh: 0.2411 mm^3^ ± 0.0137, not significant (ns) *p* > 0.9999). Figure 2(l) shows the average volume and Gundersen's coefficient of the DG blades and the KA-induced focal lesion obtained after a stereological Cavalieri analysis.

In addition to the morphological alterations described above, KA-injected mice also showed cell loss in the hilus, as well as in the CA3 and CA1 hippocampal subregions. The cell loss observed in CA1 was consistent, but of variable severity among mice. Summarizing, in KA-injected mice, we observed three main features: (1) a progressive increase of GCL widening over time, being more pronounced at 30 dpi than at 10 dpi; (2) a focal lesion in the upper blade where KA diffused; and (3) cell loss in the hilus, as well as in the CA3 and CA1 hippocampal subregions.

### 3.2. Perforant Pathway: Dentate Gyrus Long-Term Potentiation In Vivo

**Figure 3:**
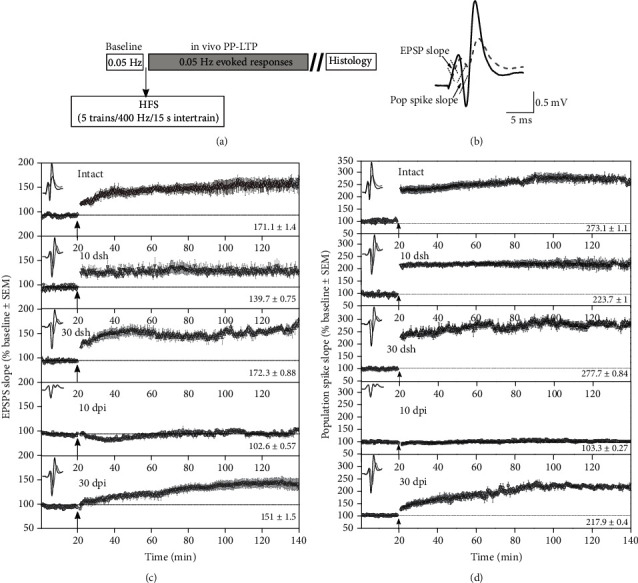
KA-induced alterations in the DG modify in vivo perforant pathway LTP along time. (a) Schematic representation of experimental procedures. Conditioning stimulus trains began after 20 min of baseline recording and were maintained for 120 min. (b) Representative traces of the excitatory postsynaptic potential (EPSP) obtained during baseline (dotted line) and 140 min after stimulation (full line). (c) Plot of the EPSP slope for the last 20 min after high-frequency stimulation. Intact and 30 dsh groups displayed similar slope percentages of EPSP (intact 171.1%; 30 dsh 172.3%). The EPSP slope percentage in the 10 dsh group was 139.7%, that is, less than those in intact and 30 dsh groups. In the 10 dpi group, LTP induction was impaired, as opposed to the 30 dpi group where LTP induction was recovered. Notably, LTP from mice at 30 dpi took longer time to reach its maximal response compared to all other groups. All these findings are more evident in the population spike slope percentage. (d) Inner numbers show the mean ± SEM of slope (percent of baseline) from the last 20 min of recording. Application of conditioning stimulus trains is indicated (arrow). Repeated measures ANOVA followed by post hoc Fisher's test; intact *n* = 4; 10 dsh *n* = 4; 30 dsh *n* = 4; 10 dpi *n* = 4; 30 dpi *n* = 4.

In order to evaluate the functional impact of KA-induced alteration on DG information processing arising from the PP, we analyzed the establishment of LTP in the perforant pathway-dentate gyrus (PP-DG) using the protocol depicted in Figure 3(a).  Figure 3(b) shows representative traces of the excitatory postsynaptic potential (EPSP) obtained during baseline (dotted line) and 140 min after stimulation (full line). After high-frequency stimulation, all groups displayed LTP, except for the 10 dpi group, in which LTP was impaired (Figures 3(c) and 3(d)). LTP was similar in intact and 30 dsh mice showing a 71 and 72% EPSP slope increase, respectively. Still, mice from the 10 dsh group showed a more discrete EPSP slope increase of only 40% without a progressive rise in magnitude along the recording (Figure 3(c)). At 30 dpi, the ability to produce LTP was restored, though with a discrete EPSP slope increase of 51%, which was lower than that observed in intact and 30 dsh groups (Figure 3(c)). Notably, LTP from 30 dpi subjects took longer to reach its maximal response compared to all other groups. The difference in the slope between the 30 dpi vs control groups (i.e. intact and 30 dsh) and 10 dsh vs control groups was statistically significant (repeated measures ANOVA differences *F*_(4, 15)_ = 133.64, *p* < 0.0001, and post hoc Fisher's test, *p* < 0.0001). We also analyzed the population spike slope change, and similar results were found as for the EPSP slope. Indeed, the trend was the same, although differences were more pronounced in the population spike slope as expected (Figure 3(d), repeated measures ANOVA differences *F*_(4, 15)_ = 549.84, *p* < 0.0001, and post hoc Fisher's test, *p* < 0.0001). Therefore, in KA-injected mice, LTP induction was impaired at 10 dpi, but at 30 dpi, the ability to elicit LTP was restored, though with a lower increase percentage than in control mice.

### 3.3. Mice at 10 dpi and 30 dpi Show Increased Locomotor Activity and Anxiety-Like Behavior

**Figure 4:**
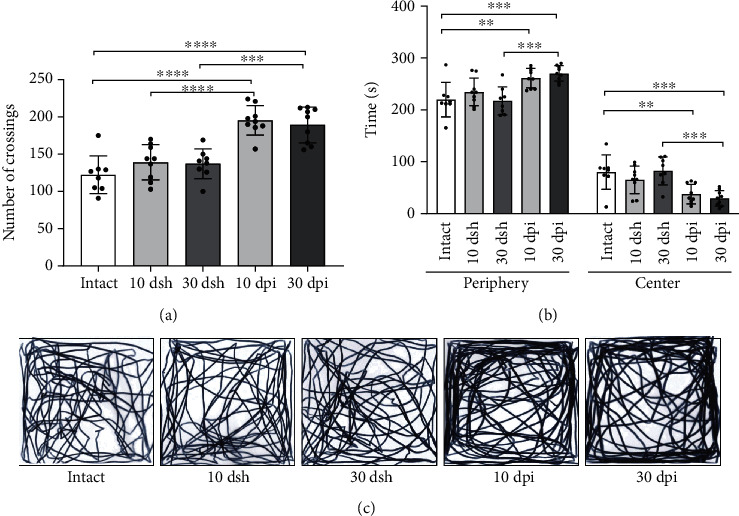
Mice at 10 dpi and 30 dpi show increased motility and anxiety-like behavior. (a) Motor behavior was evaluated through the number of crossings. KA-injected mice at 10 and 30 dpi show a higher number of crossings than intact and sham mice (one-way ANOVA followed by a Bonferroni's post hoc test, *F*_(4, 38)_ = 18.58, *p* < 0.0001). (b) Anxiety-like behavior was evaluated through the time spent in the periphery and center zones of the field. KA-injected mice at 10 and 30 dpi spent more time on periphery and less time on center zones than intact and sham mice (two-way ANOVA followed by a Bonferroni's post hoc test, interaction *F*_(4, 76)_ = 16.44, *p* < 0.0001). (c) Representative path length tracks from a mouse from each group are shown. The columns represent the mean with SD from each group. Dots superimposed in the graph represent individual values. Asterisks indicate statistically significant differences (^∗^*p* ≤ 0.05; ^∗∗^*p* < 0.01; ^∗∗∗^*p* < 0.001; ^∗∗∗∗^*p* < 0.0001). Intact *n* = 8; 10 dsh *n* = 9; 30 dsh *n* = 8; 10 dpi *n* = 9; 30 dpi *n* = 9.

Mice were evaluated in an open field task to estimate their locomotor performance and anxiety behavior. During the behavioral assessment, none of the mice displayed behavioral seizures. We observed that KA-injected mice at 10 dpi had an increased motility and anxiety-like behavior, which persisted until 30 dpi. Motility was assessed through the number of line crossings along the open field test. KA-injected groups displayed a similar number of line crossings, which was higher than in the control groups (intact and sham) (Figure 4(a); 10 dpi (mean ± SD): 195.5 ± 19.87 and 30 dpi: 189.2 ± 23.92 > intact: 122.4 ± 25.46, 10 dsh; 139.1 ± 23.62, and 30 dsh: 137.3 ± 19.9, *p* < 0.0001). Anxiety behavior was evaluated through the time spent in the periphery and center zone of the arena. KA-injected mice at 10 and 30 dpi spent more time in the periphery and less time at center zone than control mice, which reflected an anxious behavior (Figures 4(b) and 4(c); two-way ANOVA followed by a Bonferroni's post hoc test, interaction *F*_(4, 76)_ = 16.44, *p* < 0.0001). Therefore, KA-injected mice showed augmented motility and anxiety-like behavior, which lasted from 10 to 30 dpi.

### 3.4. Contextual Fear Conditioning Is Initially Impaired and Restored along Time

**Figure 5:**
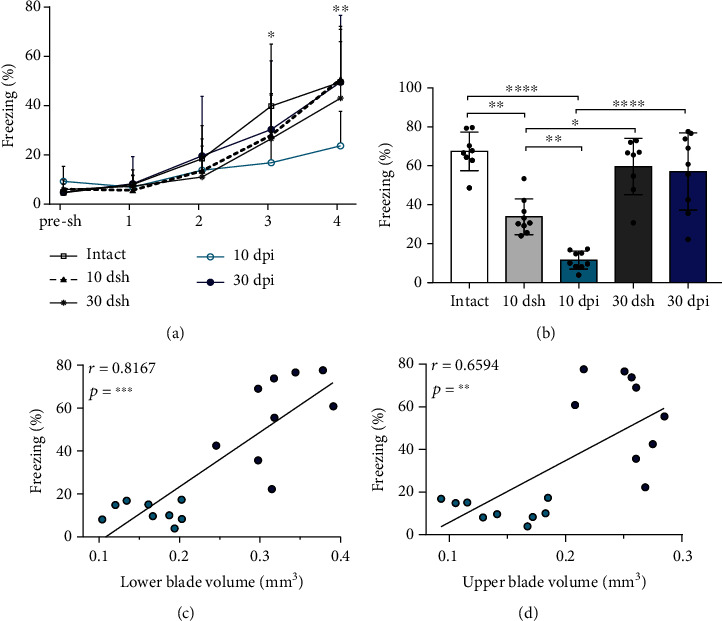
Contextual fear memory was impaired at 10 dpi but restored at 30 dpi. (a) Learning curve for contextual fear conditioning. All groups, except mice at 10 dpi, showed progressive learning reflected by the increase in the percentage of freezing time across trials. Mice at 10 dpi show lower freezing percentage time than intact mice on trials 3 and 4. Points represent the mean with SD for each group across trials (asterisks denote differences from intact vs 10 dpi; two-way ANOVA followed by a Bonferroni's post hoc test, (*F*_(4,192)_ = 43.07, *p* < 0.0001). (b) Contextual fear memory retrieval. Mice at 10 dsh show lower freezing than intact mice, but more freezing than mice at 10 dpi. At 30 dpi, mice show similar freezing as intact and 30 dsh mice. The columns represent mean and SD for each group. Dots superimposed on the graph represent individual values (one-way ANOVA followed by Bonferroni's post hoc test, *F*_(4, 38)_ = 28.29, *p* < 0.0001). (c, d) Graphs show the positive correlation between the volume of the (c) lower blade (Spearman correlation coefficient, *r* = 0.8167; 95% confidence interval (0.5552 to 0.9313); *n* = 18) and the volume of (d) the upper blade (Spearman correlation coefficient, *r* = 0.6594; 95% confidence interval (0.2644 to 0.8650); *n* = 18) with the percentage of freezing in lesioned mice. Light-blue circles correspond to 10 dpi mice (*n* = 9) and navy-blue circles to 30 dpi mice (*n* = 9). For (a, b), intact n = 8, 10 dsh *n* = 9, 30 dsh *n* = 8, 10 dpi *n* = 9, 30 dpi *n* = 9. Asterisks indicate statistically significant differences, ^∗^*p* ≤ 0.05; ^∗∗^*p* ≤ 0.01; ^∗∗∗^*p* ≤ 0.001.

We next asked if the development over time of GCL widening had a negative impact on a hippocampal-dependent behavior. To address this question, we evaluated the performance of mice in contextual fear conditioning (CFC), a hippocampus-dependent cognitive task [21, 22]. All mice, except the 10 dpi group, exhibited progressive learning during conditioning, which was reflected by the continuous increase in freezing behavior across trials (Figure 5(a); two-way ANOVA followed by a Bonferroni's post hoc test, *F*_(4,192)_ = 43.07, *p* < 0.0001). This indicates that KA-injected mice at 10 dpi, but not at 30 dpi, were impaired in this learning paradigm. Contextual fear memory was evaluated 24 h after conditioning, and the 10 dpi group displayed a lower percentage in freezing time than mice from all other groups, denoting impairment in contextual fear learning and memory (Figure 5(b); 10 dpi: 11.54 ± 4.623 < intact: 67.43 ± 9.88, 10 dsh: 33.88 ± 9.151, 30 dsh: 59.64 ± 14.59, and 30 dpi: 57.07 ± 19.78). However, at 30 dpi, KA-injected mice had increased their freezing response exhibiting values similar to intact and 30 dsh mice, which evidences a contextual fear memory recovery (Figure 5(b); 30 dpi 57.07 ± 19.78 vs intact 67.43 ± 9.88 and 30 dsh 59.64 ± 14.59, ns: *p* > 0.9999). These results are in line with our electrophysiological observations where LTP was impaired at 10 dpi and restored at 30 dpi. Concerning contextual fear memory retrieval in mice from the 10 dsh group, they displayed a diminished percentage in freezing time than mice from intact and 30 dsh (Figure 5(b); 10 dsh 33.38 ± 9.15 < intact67.43 ± 9.88, and 30 dsh 59.64 ± 14.59). The lower freezing percentage of time observed in the 10 dsh group correlated with the lower LTP response and coincides with the mechanical lesion observed in Nissl-stained tissue due to the cannula insertion. The mild impairment in LTP as well as the cannula-induced damage was completely recovered in the 30 dsh group, and the percentage in freezing time was similar to that observed in intact animals (Figure 5(b); 30 dsh: 59.64 ± 14.59 vs intact: 67.43 ± 9.88, ns: *p* > 0.9999).

Given that GCL widening increased along time, as did the freezing percentage in CFM, we evaluated if the development of GCL widening at 10 and 30 dpi correlated with the percentage in freezing time observed during memory recall. We found a positive correlation between the increase in the volume of the lower blade (reflecting GCL widening) as well as in the upper blade and the increase in freezing percentage across time (Figures 5(c) and 5(d); Spearman correlation coefficient).

Overall, these results show that the progressive GCL widening caused by excitotoxicity does not hinder the recovery of a hippocampal-dependent cognitive behavior and of LTP in the PP-DG pathway.

## 4. Discussion

In this work, we evaluated how hippocampal cognitive behavior and synaptic plasticity in the PP-DG pathway adapt in time along GCL widening due to a focal, unilateral injection of KA in the upper blade of the DG. We found that the gradual increase in GCL widening did not associate with a sustained deficit in cognitive behavior or with impaired synaptic plasticity along time. Compromised cognitive behavior and LTP in the PP-DG pathway were observed to occur along a mild increase in GCL widening at 10 days post-KA injection (dpi). However, the recovery of cognitive behavior and improved LTP occurred despite a pronounced increase in GCL widening at 30 dpi.

We found that after inducing a KA focal lesion in the DG, a similar progressive development of GCL widening occurred as previously reported in other studies where widening began to be visible after 1 week of KA injection and reached its peak after 3 weeks [23, 24]. This time window allowed us to study the impact of GCL widening development in the DG function: at 10 dpi when the widening was readily visible and at 30 dpi when it was fully developed. Our results provide evidence that the DG reinstates some of its intrinsic functions despite GCL widening. The present work differs from studies using the KA mouse epilepsy model, where KA is injected close to the hippocampal fissure to induce complete GCD on both DG blades [25]. In our study, the lower blade (which did not receive the focal lesion) disperses more evidently than the upper blade, where the focal lesion was induced. In this regard, the upper blade showed a slight dispersion mainly in the surroundings of the focal lesion where abundant cell loss was found. An important finding in our study was that GCL widening, due to granule cell dispersion (GCD), did not impede the recovery of the hippocampal-dependent cognitive behavior nor did it block LTP in the PP-DG pathway.

It has been suggested that the progressive increase in GCL widening due to GCD triggers homeostatic changes in granule cells that contribute to maintain DG-associated functions under pathological hyperexcitation conditions [14]. In the context of hyperexcitation, electrophysiological studies report that granule cells surviving in GCD regions reduce their intrinsic excitability by decreasing calbindin-dependent calcium influx and increasing potassium outflux in both mesial temporal lobe epilepsy (MTLE) patients and in the KA mouse model of MTLE [12, 13, 26]. The rescaling in the excitability of granule cells points to a mechanism that could gradually decrease hyperexcitability in GCD zones [13]. Notably, a computational analysis suggests that this intrinsic rescaling assures the sparseness of granule cell activation and thereby maintains the associated cognitive function of the DG network after an excitotoxic insult [14]. These homeostatic changes in granule cells may be involved in the cognitive behavior improvement observed in our study at 30 dpi.

In line with the above, we observed that the pronounced GCL widening found at 30 dpi correlated in time with an improvement in LTP. One possible mechanism related to the latter is the observed strengthening of synaptic excitatory inputs to dentate granule cells from the entorhinal cortex along time after KA administration [24]. It has been shown that new mature excitatory synapses are established between granule cell dendrites and medial perforant path fibers in KA-injected mice. Spine density on the dendritic segment within the middle molecular layer, where medial perforant fibers arrive, increases over 60%. At an ultrastructural level, the presynaptic boutons of medial perforant synapses and postsynaptic spines of granule cells are enlarged, an ultrastructural feature reminiscent of LTP-like plasticity [24]. Another possible mechanism impacting LTP increment is the gradual increase of brain-derived neurotrophic factor (BDNF) mRNA in granule cells along with granule cell dispersion development. Notably, in KA mice, a positive correlation between BDNF mRNA levels and granule cell dispersion development has been reported [9]. It has been shown that BDNF is critically involved in the induction and maintenance of LTP [27–29]. In line with this, it has been observed that granule cells in GCD regions display a robust increase in evoked excitatory postsynaptic current (whole-cell recording) during extracellular stimulation of the medial perforant path [24].

It should be also considered that the low activity of granule cells is important for the proper performance of DG-associated network function and hippocampal cognition [4]. This low activity is maintained in part through the strong inhibition exerted by GABAergic interneurons in the DG [30]. During the first week after KA administration, studies report a reduction in the inhibitory efficacy in the DG. The levels of GABA decrease early at day 1 after the intrahippocampal KA administration in mice and remain low within the first week [31]. Moreover, the E_GABA_ (current equilibrium potential) of granule cells shifts to less hyperpolarized levels (−55 to −60 mV) during the first week after excitotoxic damage [32], which diminishes inhibitory efficacy while increasing excitability in granule cells. Interestingly, the levels of GABA return to normal at four weeks after the intrahippocampal KA administration in mice [24]. Similarly, E_GABA_ in granule cells return to near control levels at four weeks after pilocarpine administration, which increases inhibitory efficacy in DG [32]. The restoration of inhibition in the DG is important for the establishment of proper excitation/inhibition balance. Given that restoration occurs around week four, it is possible to suggest that it contributes to the reestablishment of LTP and to the cognitive recovery observed at 30 dpi in our study.

Our electrophysiological results show that despite the visible improvement in LTP at 30 dpi, the trace differed from that in the intact and 30 dsh groups. LTP at 30 dpi shows a lower response and a slower increase across time after high-frequency stimulation (HFS). Therefore, it is likely that the mechanisms related to the early-LTP phase induction are impaired in mice at 30 dpi. These mechanisms involving the PP-DG pathway associate with the activation of AMPA and NMDA receptors [33, 34]. In the KA mouse model, the density of AMPA receptors is attenuated in dispersed zones, which predicts a weakening of synaptic efficacy [35]. The low density of AMPA receptors may delay the entry of Ca^2+^ necessary to induce the fast increase in early-LTP response [33]. Consequently, other mechanisms like activation of metabotropic receptors and subsequent increase of cAMP may be responsible for the slow increase in LTP observed here at 30 dpi [36]. Further studies are necessary to reveal the causes that underlie this different LTP response. However, this suggests that synaptic plasticity in the PP-DG pathway is still compromised at 30 dpi, but with a visible improvement compared to 10 dpi.

Results from the 10 dsh group show a lower LTP response and freezing percentage time during memory retrieval compared to control mice (i.e. intact and 30 dsh). We associate these events with the interruption of the granule cell layer observed by the cannula insertion during surgery procedures. It is likely that this mechanical lesion promoted the activation of calpains, which are increased in neuronal damage resulting from traumatic injury [37]. Calpains can limit the magnitude of LTP and compromise cognition [38]. Likewise, mice from the 10 dpi group may have been affected by the cannula insertion. However, this group showed a complete impairment in both evaluated DG functions reflecting the contribution of KA-induced excitotoxic damage.

In our study, we evaluated contextual fear learning and memory after hippocampal damage and show that at 10 dpi, contextual fear learning and memory are compromised. However, at 30 dpi, learning and memory show to be reinstated (as shown by the increase in freezing time). It is feasible that at 10 dpi, the DG is still under a high imbalance that prevents animals from forming new episodic memories due for instance to altered input from the entorhinal cortex [24], loss of intrinsic connections, reduction in the inhibitory efficacy [24], increased network excitability [5], or astrogliosis [39] among others. However, at 30 dpi, the DG may have compensated for the above-suggested disruptions even in the presence of GCD. Therefore, the fact that we observed anterograde amnesia at an early but not at a later time point after damage may be related to reorganization of the remaining tissue as well as to plastic mechanisms leading to the return to homeostasis in the affected structure and its circuitry. Alternatively, it is possible to suggest that in the presence of a long-lasting damaged DG, extrahippocampal structures (i.e., neocortex) are recruited for context processing [40] or that KA infusion provoked alterations in extrahippocampal regions which may influence the early impairment and later recovery of the task. In line with the latter, a marked loss of projections from the hippocampus to the retrosplenial cortex and the mammillary bodies following dorsal hippocampus KA injections has been reported [41]. As hippocampal afferents to such structures are involved in memory processing, both structures have been implicated in the encoding of contextual fear conditioning [42, 43].

Our results show that KA-lesioned mice display anxiety and hyperactive phenotypes in the open field test. This is in line with previous results showing that anxiety and hyperactive behaviors are still observed after one month of KA injection [44]. Moreover, the anxiogenic behavior observed in our KA-injected mice could also be explained by the expansion of GCD in more temporal sections (Supplementary Figure 1, 2 and 3). Previous studies have shown that hippocampal lesions, optogenetic modulation of granule cell activity, and directly induced GCD in this DG zone can provoke these behaviors in rodents [44–46]. However, more precise anxiety behavioral tests would help further characterize the anxiogenic response in our experimental animals.

Finally, one possible caveat in our learning and memory results is that lesioned mice show a hyperactive phenotype in the open field test; it could be argued that this would complicate the interpretation on the freezing results observed in the memory task. However, while both groups, 10 and 30 dpi, exhibited similar hyperactive behavior, only mice from the 10 dpi group displayed learning and memory impairments. This supports the conclusion that hyperactive behaviors might not account for the cognitive results observed here. However, two considerations could emerge to interpret the result concerning the lower freezing of the 10 dpi group. First, this group may undergo a transient change in noxious responding to the foot shock resulting in less learning and hence lower freezing. In this line, it is to highlight that we observed similar mice reactivity to the foot shocks among all groups, which were reflected by jumps and vocalizations. The latter suggests no change in noxious response to the foot shocks occurred at any time point. Second, it may be possible that mice from the 10 dpi group displayed an active avoidance strategy, leading to reduced freezing during conditioning. However, this seems unlikely for us given that 30 dpi animals, which exhibit similar hypermotility, did not display an active avoidance strategy during the learning phase.

In conclusion, our results show that GCL widening does not hinder long-term DG function at a behavioral nor at a synaptic level, and together with evidence provided from other groups, our results may contribute to the idea that injury-induced GCL widening following excitotoxicity is not enough to impede DG functions over time.

## Data Availability

Data are available through direct request to the corresponding authors.

## Supplementary Materials

Supplementary 1 Supplementary Figure 1: GCD through the septotemporal axis. Images show representative Nissl-stained coronal sections of the hippocampus from each group: (A) sham, (B) 10 dpi, and (C) 30 dpi. Sham mice did not develop GCD. At 10 and 30 dpi, mice developed GCD in both blades in septal coordinates (~AP: −2.06 to −2.54 mm from the bregma) and in most temporal coordinates (~ AP: −3.16 to −3.52 mm from bregma). GCD is visible only in the top part of the DG. Note that GCD is more severe at 30 dpi than 10 dpi in the septal and temporal coordinates. Scale bar: 300 *μ*m.

Supplementary 2 Supplementary Figure 2: GCD is present in the top (arrow) but not in the bottom (head arrow) part of the DG in the most posterior sections (~AP: −3.16 to −3.52 mm, and ~DV: −2.12 to −2.75 mm). Scale bar: 300 *μ*m.

Supplementary 3 Supplementary Figure 3: GCD develops in the injected but not in the contralateral DG. Arrows indicate the top part of the DG displaying GCD. Scale bar: 300 *μ*m.

Supplementary 4 Graphical abstract of this article. Progression of DG structural alterations occurs along functional recovery.
