# Supplementary figures and images for: Progression in Time of Dentate Gyrus Granule Cell Layer Widening due to Excitotoxicity Occurs along In Vivo LTP Reinstatement and Contextual Fear Memory Recovery
Source: Neural Plast. 2022 Sep 27;2022:7432842. doi: 10.1155/2022/7432842 (PMC9533134; doi:10.1155/2022/7432842)

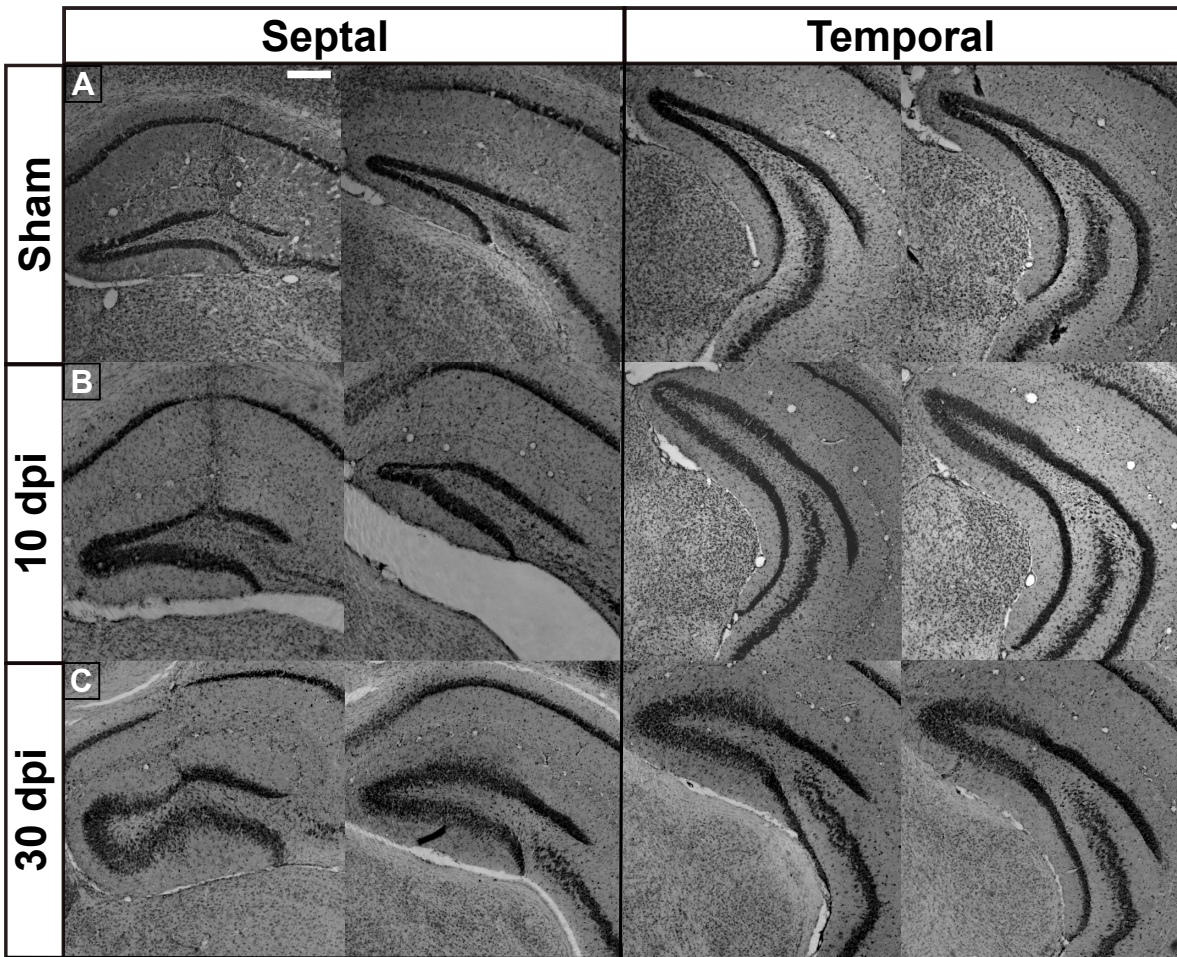

Supplement: Supplementary 1 — Supplementary Figure 1: GCD through the septotemporal axis. Images show representative Nissl-stained coronal sections of the hippocampus from each group: (A) sham, (B) 10 dpi, and (C) 30 dpi. Sham mice did not develop GCD. At 10 and 30 dpi, mice developed GCD in both blades in septal coordinates (~AP: −2.06 to −2.54 mm from the bregma) and in most temporal coordinates (~ AP: −3.16 to −3.52 mm from bregma). GCD is visible only in the top part of the DG. Note that GCD is more severe at 30 dpi than 10 dpi in the septal and temporal coordinates. Scale bar: 300 μm. [file 7432842.f1.pdf]

**Sham**

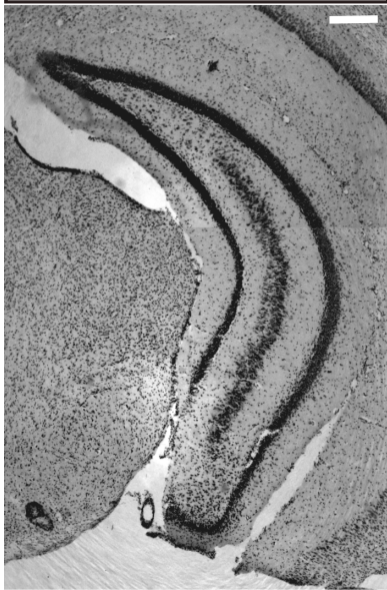

**10 dpi**

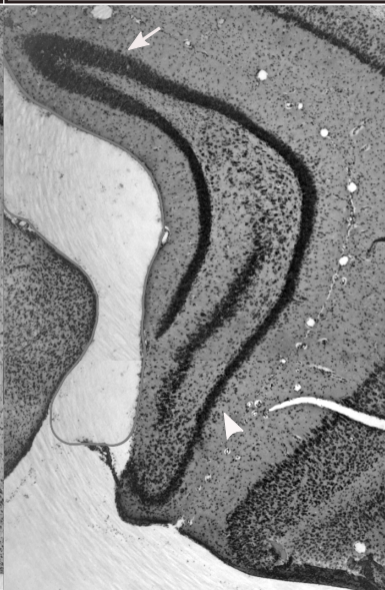

**30 dpi**

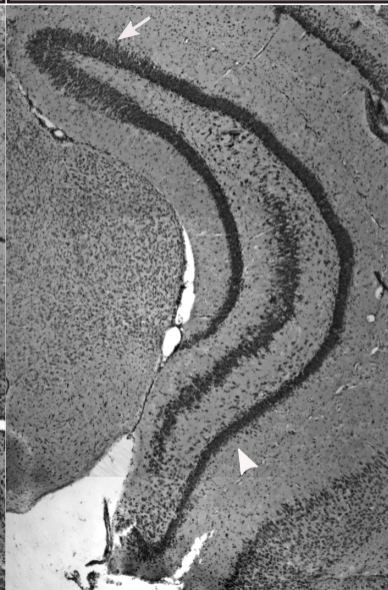

Supplement: Supplementary 2 — Supplementary Figure 2: GCD is present in the top (arrow) but not in the bottom (head arrow) part of the DG in the most posterior sections (~AP: −3.16 to −3.52 mm, and ~DV: −2.12 to −2.75 mm). Scale bar: 300 μm. [file 7432842.f2.pdf]

**Contralateral**

**Ipsilateral**

**10 dpi**

**30 dpi**

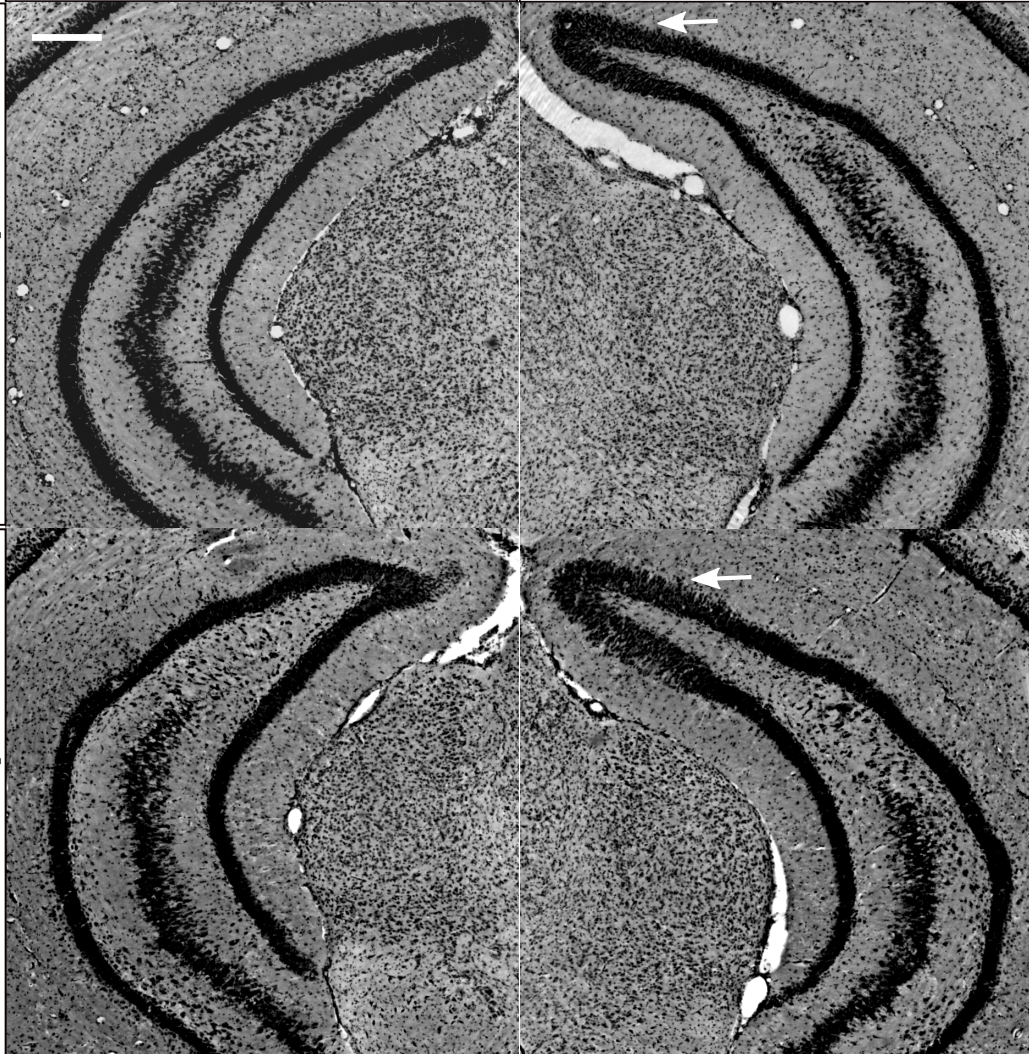

Supplement: Supplementary 3 — Supplementary Figure 3: GCD develops in the injected but not in the contralateral DG. Arrows indicate the top part of the DG displaying GCD. Scale bar: 300 μm. [file 7432842.f3.pdf]
