# Supplementary material for: Progression in Time of Dentate Gyrus Granule Cell Layer Widening due to Excitotoxicity Occurs along In Vivo LTP Reinstatement and Contextual Fear Memory Recovery
Source: Neural Plast. 2022 Sep 27;2022:7432842. doi: 10.1155/2022/7432842 (PMC9533134; doi:10.1155/2022/7432842)

Graphical Abstrac: Progression of DG structural alterations occur along functional recovery

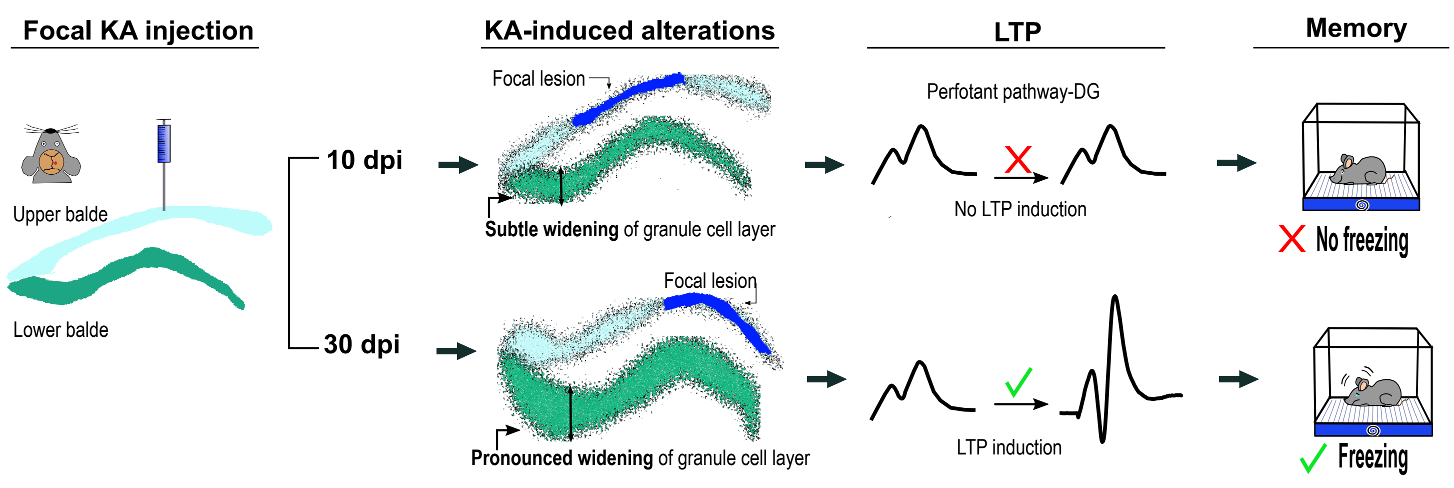

Supplement: Supplementary 4 — Graphical abstract of this article. Progression of DG structural alterations occurs along functional recovery. [file 7432842.f4.pdf]
